# Comparative Analysis of Machine Learning Algorithms on Surface Enhanced Raman Spectra of Clinical *Staphylococcus* Species

**DOI:** 10.3389/fmicb.2021.696921

**Published:** 2021-08-31

**Authors:** Jia-Wei Tang, Qing-Hua Liu, Xiao-Cong Yin, Ya-Cheng Pan, Peng-Bo Wen, Xin Liu, Xing-Xing Kang, Bing Gu, Zuo-Bin Zhu, Liang Wang

**Affiliations:** ^1^Department of Bioinformatics, School of Medical Informatics and Engineering, Xuzhou Medical University, Xuzhou, China; ^2^State Key Laboratory of Quality Research in Chinese Medicines, Macau University of Science and Technology, Taipa, China; ^3^Department of Laboratory Medicine, School of Medical Technology, Xuzhou Medical University, Xuzhou, China; ^4^School of Life Science, Xuzhou Medical University, Xuzhou, China; ^5^Department of Laboratory Medicine, Guangdong Provincial People’s Hospital, Guangdong Academy of Medical Sciences, Guangzhou, China; ^6^Jiangsu Key Laboratory of New Drug Research and Clinical Pharmacy, School of Pharmacy, Xuzhou Medical University, Xuzhou, China

**Keywords:** Raman spectroscopy, surface enhanced Raman spectroscopy, convolutional neural network, long short-term memory neural network, machine learning

## Abstract

Raman spectroscopy (RS) is a widely used analytical technique based on the detection of molecular vibrations in a defined system, which generates Raman spectra that contain unique and highly resolved fingerprints of the system. However, the low intensity of normal Raman scattering effect greatly hinders its application. Recently, the newly emerged surface enhanced Raman spectroscopy (SERS) technique overcomes the problem by mixing metal nanoparticles such as gold and silver with samples, which greatly enhances signal intensity of Raman effects by orders of magnitudes when compared with regular RS. In clinical and research laboratories, SERS provides a great potential for fast, sensitive, label-free, and non-destructive microbial detection and identification with the assistance of appropriate machine learning (ML) algorithms. However, choosing an appropriate algorithm for a specific group of bacterial species remains challenging, because with the large volumes of data generated during SERS analysis not all algorithms could achieve a relatively high accuracy. In this study, we compared three unsupervised machine learning methods and 10 supervised machine learning methods, respectively, on 2,752 SERS spectra from 117 *Staphylococcus* strains belonging to nine clinically important *Staphylococcus* species in order to test the capacity of different machine learning methods for bacterial rapid differentiation and accurate prediction. According to the results, density-based spatial clustering of applications with noise (DBSCAN) showed the best clustering capacity (Rand index 0.9733) while convolutional neural network (CNN) topped all other supervised machine learning methods as the best model for predicting *Staphylococcus* species *via* SERS spectra (ACC 98.21%, AUC 99.93%). Taken together, this study shows that machine learning methods are capable of distinguishing closely related *Staphylococcus* species and therefore have great application potentials for bacterial pathogen diagnosis in clinical settings.

## Introduction

The genus *Staphylococcus* includes many commonly encountered and clinically important pathogenic species in nosocomial infections, such as *Staphylococcus aureus* and *Staphylococcus epidermidis*, etc. (McGavin and Heinrichs, 2012). Some of these *Staphylococcus* species could cause severe infectious diseases, especially in immune-compromised patients with the use of catheters and other medical implants (Schlievert et al., 2016). Therefore, it is crucial to develop rapid diagnostic methods for pathogenic bacteria. Raman spectroscopy (RS) is a widely used non-destructive, vibrational spectroscopic technique in the fields of biology and medicine, such as cell-drug interactions (Buckley and Ryder, 2017) and cancer diagnosis (D’Acunto et al., 2020), etc., which normally generates spectra of the analytes that can be further used for quantitative and qualitative analyses (Das and Agrawal, 2011). The basic principle of Raman spectroscopy relies on the photons in elastically scattered after interacting with vibrating molecules within the sample. Since molecular vibrations are distinct for each molecule, the vibrational Raman spectrum for a sample is therefore unique with characteristic peaks that are often termed as molecular fingerprints. However, the major drawback for traditional RS is its inherent weakness of signals, hence very low detection sensitivity (Jones et al., 2019). In addition, it is very difficult for RS to obtain reliable spectra due to its comparatively poor reproducibility (Dong and Zhao, 2017).

Recently, enhanced Raman spectroscopic techniques have emerged, such as surface enhanced Raman spectroscopy (SERS) and tip-enhanced Raman scattering (TERS; Jones et al., 2019). SERS is a surface-sensitive technique that can enhance the intensity of Raman scattering at the level of several orders of magnitude by exploiting surface plasmons (SPs) of metallic nanostructures (Pérez-Jiménez et al., 2020), also known as SERS substrate, which is sufficient to analyze bacterial samples at single-cell resolution (Weiss et al., 2019). Common examples for SERS substrates include silver and gold nanoparticles (NPs) since they do not have any Raman active modes (Bora, 2018) and show outstanding SERS enhancements (Pérez-Jiménez et al., 2020). Comparatively speaking, silver colloidal nanoparticles (AgNPs) has a high molar extinction coefficient from visible to near infrared region, whereas gold is commonly used for red and near infrared regions; in addition, AgNPs show higher plasmon quality than that of gold NPs (Wei et al., 2018; Pérez-Jiménez et al., 2020). Therefore, AgNPs have been widely employed for bacteria detection in SERS studies, which are also the case in this study for the differentiation and identification of *Staphylococcus* species.

Due to the complexity of bacterial composition, large datasets are regularly acquired during SERS analyses, which make the classical linear methods no longer sufficient for data processing (Lussier et al., 2020). Machine learning (ML) methods focus on constructing models *via* learning patterns from large sets of data and improving the accuracy of models over time, which belongs to the field of artificial intelligence (AI). ML algorithms have been successfully applied in classification, clustering, and prediction tasks over large, high-dimensional datasets (Marsland, 2014). In fact, ML algorithms have found many applications in Raman spectroscopy, especially for the differentiation and identification of bacterial pathogens (Rebrošová et al., 2017; Chen et al., 2019; Ho et al., 2019; Uysal Ciloglu et al., 2020). For example, Rebrošová et al. (2017) recruited three classical supervised learning methods, linear discriminant analysis (LDA), one nearest neighbor (1NN), and support vector machine (SVM), to analyze 16 *Staphylococcus* strains, according to which, 1NN achieved the highest accuracy (99.3%). In addition, Ho et al. (2019) used the state-of-the art deep learning model convolutional neural network (CNN) to address low signal-noise-ratio (SNR) one-dimensional Raman spectral data for the first time, which not only achieved more than 82% prediction accuracy for bacterial identification, but also successfully differentiated methicillin-resistant (MRSA) and methicillin-susceptible *S. aureus* (MSSA) with 89±0.1% accuracy.

Currently, there is little study focusing on the systematic comparison of performances of different machine learning methods in terms of both supervised and unsupervised learning algorithms. Here, we applied three unsupervised learning algorithms and 10 supervised learning algorithms to analyze 2,752 Raman spectra generated from 117 *Staphylococcus* strains belonging to nine *Staphylococcus* species. According to the comparative study of the three unsupervised learning methods, density-based spatial clustering of applications with noise (DBSCAN) had the best capacity for clustering *Staphylococcus* species into different groups (Rand index 0.9733). We also compared the prediction capacity of 10 supervised learning algorithms, which showed that CNN was the best predicting model for analyzing *Staphylococcus* Raman spectra with accuracy (ACC) at 98.21% and area under curve (AUC) at 99.93%. Taken together, we concluded that machine learning methods were efficient for the differentiation and identification of pathogenic *Staphylococcus* species, which showed promising potentials for rapid and non-invasive clinical diagnostics of bacterial pathogens in near future.

## Materials and Methods

### Chemical and Biological Materials

A total of 117 *Staphylococcus* strains belonging to nine *Staphylococcus* species were included in this experiment: 12 strains of *S. aureus* (*N*=531), 12 strains of *S. capitis* (*N*=282), 30 strains of *S. epidermidis* (*N*=649), 18 strains of *S. haemolyticus* (*N*=360), 20 strains of *S. hominis* (*N*=550), six strains of *S. kloosii* (*N*=80), three strains of *S. sciuri* (*N*=70), eight strains of *S. warneri* (*N*=140), and eight strains of *S. xylose* (*N*=90). A total of 2,752 surface enhanced Raman spectra were collected, which was denoted by the letter *N* within the parentheses for each species. All the strains were clinical isolates stored in the Department of Laboratory Medicine, the Affiliated Hospital of Xuzhou Medical University, Xuzhou, Jiangsu Province, China. All of the strains were identified and confirmed through biochemical methods plus Matrix-assisted laser desorption/ionization-time of flight (MALDI-TOF) mass spectrometry (MS) and stored in Thermo-Fisher freezer at −80°C. Before Raman spectroscopy, all the strains were thawed, inoculated onto Mueller-Hinton agar plates (Sigma-Aldrich), and cultivated for 24h at 37°C. Colonies were randomly selected and mixed with negatively-charged silver nanoparticle substrate for SERS.

### Preparation of Negatively-Charged Silver Nanoparticle Substrate

About 200ml of deionized water (ddH_2_O) and 33.72mg of AgNO_3_ (Sinopharm, Beijing, China) was added to a clean and sterile Erlenmeyer flask, which was then gently mixed and heated on a magnetic stirrer. After boiling, 8ml of 1wt% sodium citrate was added into the mixture, which was heated for 15min at the stirring rate of 650r/min. Stop heating, continue stirring, and wait for the mixture to cool down to room temperature. The final volume was set to 200ml. Then, take 1ml of the final solution and place it in a sterile Eppendorf tube, centrifuge the tube at 7,000r/min for 7min, discard the supernatant after centrifugation, and resuspend the solution with 100μl of ddH_2_O to obtain a uniform milky gray solution. The solution is the negatively-charged silver nanoparticle substrate. Store the solution in the dark at room temperature for later use.

### Surface-Enhanced Raman Spectroscopy

After cultivation, a single colony of a *Staphylococcus* species was inoculated into 15μl phosphate buffer saline (PBS) and well mixed *via* vigorous vortexing, which was then well mixed with 15μl negatively-charged silver nanoparticle substrate solution. The mixed solution was dropped onto silicon wafer for complete dry. The dried spot was then measured by commercial i-Raman® Plus Raman spectrometer BWS465-785H (B&W Tek, United States) for Raman spectral generation. Measurement settings were described below. Laser power: 340mW, nominal at exiting probe; 455mW, nominal at laser port. Wavelength: 785nm. Detector type: high quantum efficiency CCD array. Raman shift range: 65–2,800cm^−1^. Spectral acquisition: 20s. Resolution: <3.5cm^−1^ at 912nm. Each spectrum consists of 657 points measured in the range 519.56–1,800.81cm^−1^.

### Preliminary Analysis of Raman Spectra

#### Averaged Raman Spectra

Original data for each sample were sourced from Raman spectrometer *via* the software BWSpec 4.02 (B&W Tek, United States) and saved in plain text format. For all spectral files in a *Staphylococcus* species, the columns Raman shift and Raman intensity were first extracted from 519.56 to 1,800.81cm^−1^ and then put together *via* in-house Python scripts. The re-organized data were further calculated for average intensity and standard deviation at each Raman shift and visualized *via* Origin (OriginLab, United States).

#### Identification of Characteristic Peaks

The software LabSpec 6 (HORIBA Scientific, Japan) was used for processing and smoothing the averaged Raman spectra data. The parameters were first set at Degree=4, Size=5, and Height=50, and then click the button “Smooth.” For baseline correction, use the following settings: Type=Polynom, Degree=6, Attach=No, and then click the button “Auto.” After that, start to search the characteristic peaks. Function was set to GaussLoren, Level to 13%, and Size to 19 while other parameters were kept in default. Then, click on the “Search” button. Finally, use LabSpec 6 to normalize the spectra in order to better compare the curves from different *Staphylococcus* species. All the characteristic peaks were annotated with a black arrow. Common biopolymers, such as nucleic acids, proteins, lipids, and carbohydrates, etc. have been widely studied by Raman spectroscopy, which has led to the assignment of the Raman characteristic peaks to various molecular vibrations as summarized in Table 1. Dot matrix plot was also drawn to visualize the distribution of characteristic peaks among the nine *Staphylococcus* species in Supplementary Figure 1.

**Table 1:** Band assignments of characteristic peaks to potential metabolites in Raman spectra of *Staphylococcus* species.

Raman shift (cm^−1^)	Band assignment	References
555–562	Guanine/Thymine/Uridine	Mert et al., 2015
649–654	Guanine	Ahmed et al., 2013
727/730/732	Nucleic acids	Chao and Zhang, 2012
856	Tyrosine	Chaturvedi et al., 2016
957/958	C=C	Ahmed et al., 2013
1,003	C-H	Chaturvedi et al., 2016
1,048	P-O	Chen et al., 2015
1,089/1,093	Phenylalanine	Ahmed et al., 2013
1,242	Amide III	Chisanga et al., 2018
1,323–1,330	Adenine ring	Chisanga et al., 2018
1,370–1,383	Amide III	Perez-Guaita et al., 2016
1,445–1,466	N=N aromatic and aliphatic	Nguyen et al., 2013
1,577–1,582	Guanine/Adenine	Chisanga et al., 2018
1,689–1,697	C=O, C=C	Nguyen et al., 2013

### Machine Learning Methods

#### Data Preprocessing

Principal component analysis (PCA) was used to reduce the dimensionality of each *Staphylococcus* Raman spectra. According to PCA analysis, a few meaningful dimensions were identified, which were mainly determined by the degree of dispersion (variance) of all observations in each dimension. Total variance contribution rate (≥99%) was used as an indicator in this study. The results showed that 10 principal components were found. In order to avoid different units from affecting the results of data analysis, all data were normalized by column to improve the accuracy and accelerate the convergence speed of subsequent supervised and unsupervised machine learning algorithms.

#### Unsupervised Learning

Three clustering algorithms, K-means clustering algorithm (K-means), agglomerative nesting (AGNES), and DBSCAN, were used in this study to analyze the pre-processed Raman spectral data *via* PCA. In particular, we set the *K* value (*n*_clusters) in the K-means algorithm to 9, and divided each point into the cluster represented by the nearest cluster center point. After all points were allocated, these points in the cluster were re-calculated in terms of the center point of the cluster by taking the average value. Thus, the center point of the cluster was iteratively re-allocated and updated until the center point of the cluster changed little or reached the specified iteration frequency. As for the AGNES hierarchical clustering algorithm, we also set the *K* value (*n*_clusters) to 9, and the linkage mode was set to “ward,” that is, minimization of the differences in all clusters in terms of sum of squares. By using the bottom-up strategy, each object was initially treated as a cluster. Then, these atomic clusters were merged into a larger cluster until all objects were in the same cluster or met the termination condition. In terms of DBSCAN algorithm, the minimum rough value (min_samples) was set to 9, and the density radius was set to 0.7. By using the scikit-learn library in Python (Pedregosa et al., 2011), we calculated the adjusted Rand Index with a value between [−1, 1] so as to measure the degree of agreement between the clustering results and the real situation. The closer the value is to 1, the better the clustering effect is.

#### Supervised Learning

**Figure 1:**
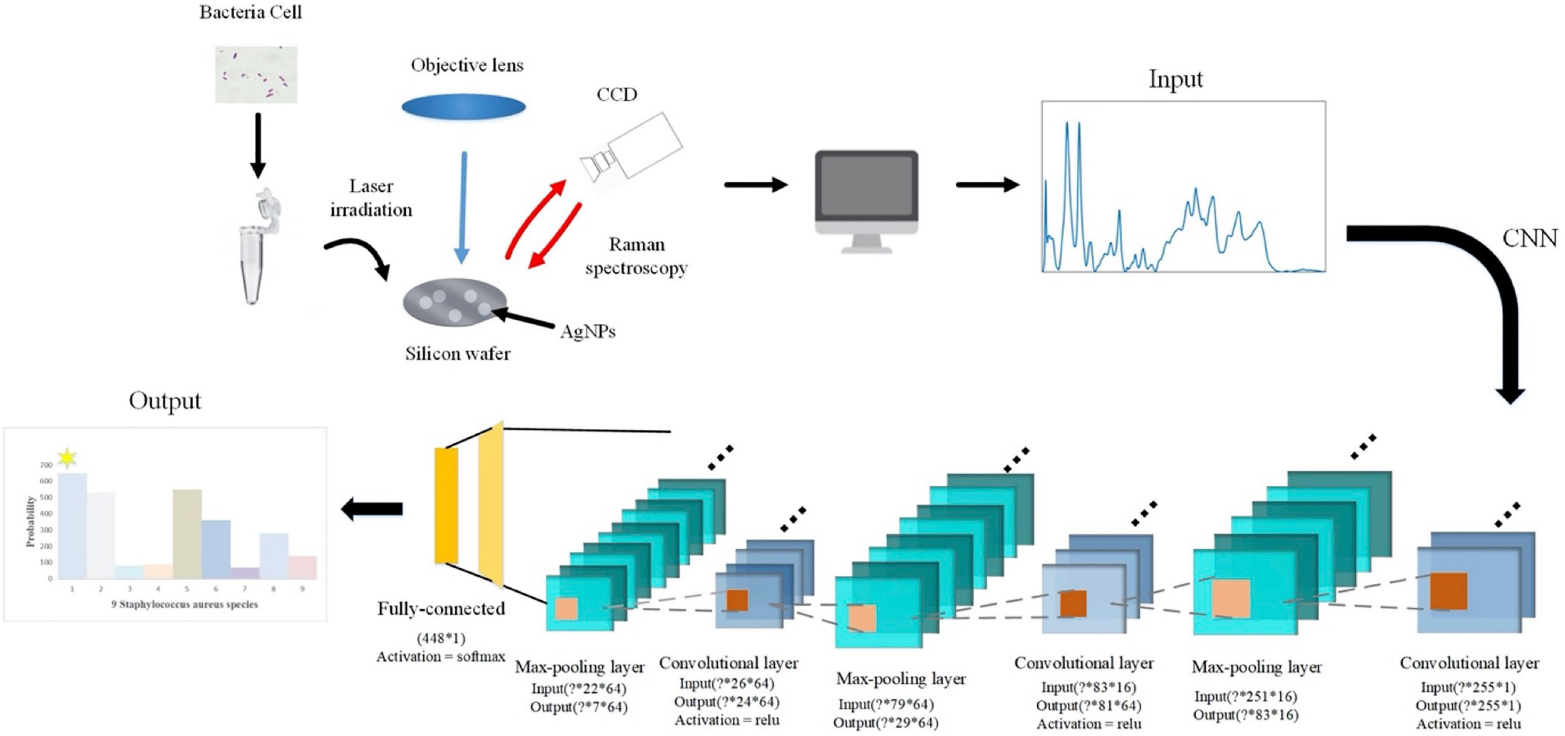
Schematic illustration of convolutional neural network (CNN) data flow during processing surface enhanced Raman spectra of *Staphylococcus* species. In specificity, a particular strain was first mixed with silver colloidal nanoparticles (AgNPs) and then smeared on the silicon chip. Raman spectrum fingerprinting data in the smearing area were then generated. In-house python scripts were used to perform principal component analysis (PCA) for dimension reduction and spectral normalization, which was then processed through alternating convolutional layers and pooling layers *via* different activation functions in order to classify and predict the nine *Staphylococcus* species.

According to the spectral characteristics of *Staphylococcus* species, we used eight types of traditional supervised machine learning methods that are K-nearest neighbors (KNN, KNeighbors), decision trees (DT, DecisionTree), random forest (RF, RandomForest), gradient boosting (GB, GradientBoosting), SVM, adaptive boosting (AdaBoost), Gaussian naive Bayes (GNB, GaussianNB), quadratic discriminant analysis (QDA, QuadraticDiscriminantAnalysis), and two deep learning methods, namely, CNN and long short-term memory neural network (LSTM) to process one-dimensional Raman spectral fingerprinting data *via* scikit-learn library (Pedregosa et al., 2011). For all the algorithms, the sample data were divided into 70% of the training set and 30% of the test set. We then converted previously defined labels into a hot encoding form that could be easily recognized by the computer. Encapsulated classifier functions in the scikit-learn library were called for the analysis while the corresponding parameters were set, accordingly. For example, we set the kernel function of SVM to *rfb*, the penalty parameter *C*=0.8, and the kernel function parameter gamma=20. As for the two deep learning algorithms, CNN consisted of one initial input layer, three alternative convolutional layers, and three pooling layers, plus one fully connected layer and output layer (Figure 1). The three convolutional layers contained 16, 64, and 64 convolution filters of different sizes. A single training iteration (epochs) was set to 5, the number of batches (batch_size) for each training was set to 10, and *adam* was selected for the optimization algorithm of the loss function.

**Figure 2:**
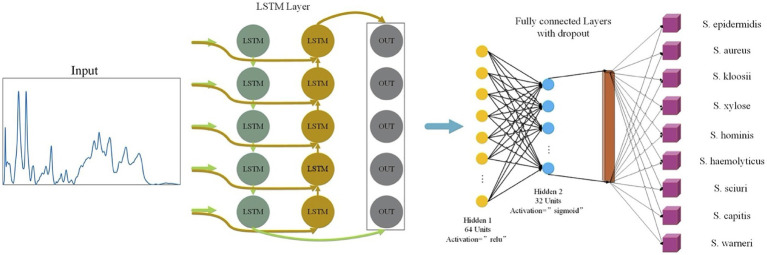
Schematic illustration of long short-term memory neural network (LSTM) analysis of *Staphylococcus* Raman spectra. The one-dimensional Raman spectra data were first normalized and then used as input for LSTM. After a fully connected layer and regularization operation, different activation functions were used to improve the accuracy of the model. After going through a fully connected neural layer, Raman spectral data of *Staphylococcus* species were correspondingly classified and predicted.

In this study, LSTM contained an input layer, two hidden layers, two regularization methods, and a fully-linked neural layer. The two hidden layers used the *relu* and *sigmoid* activation functions, respectively (Figure 2). The optimization algorithm used for the loss function was *adam* while the single training iteration (epochs) was set to 50.

**Figure 3:**
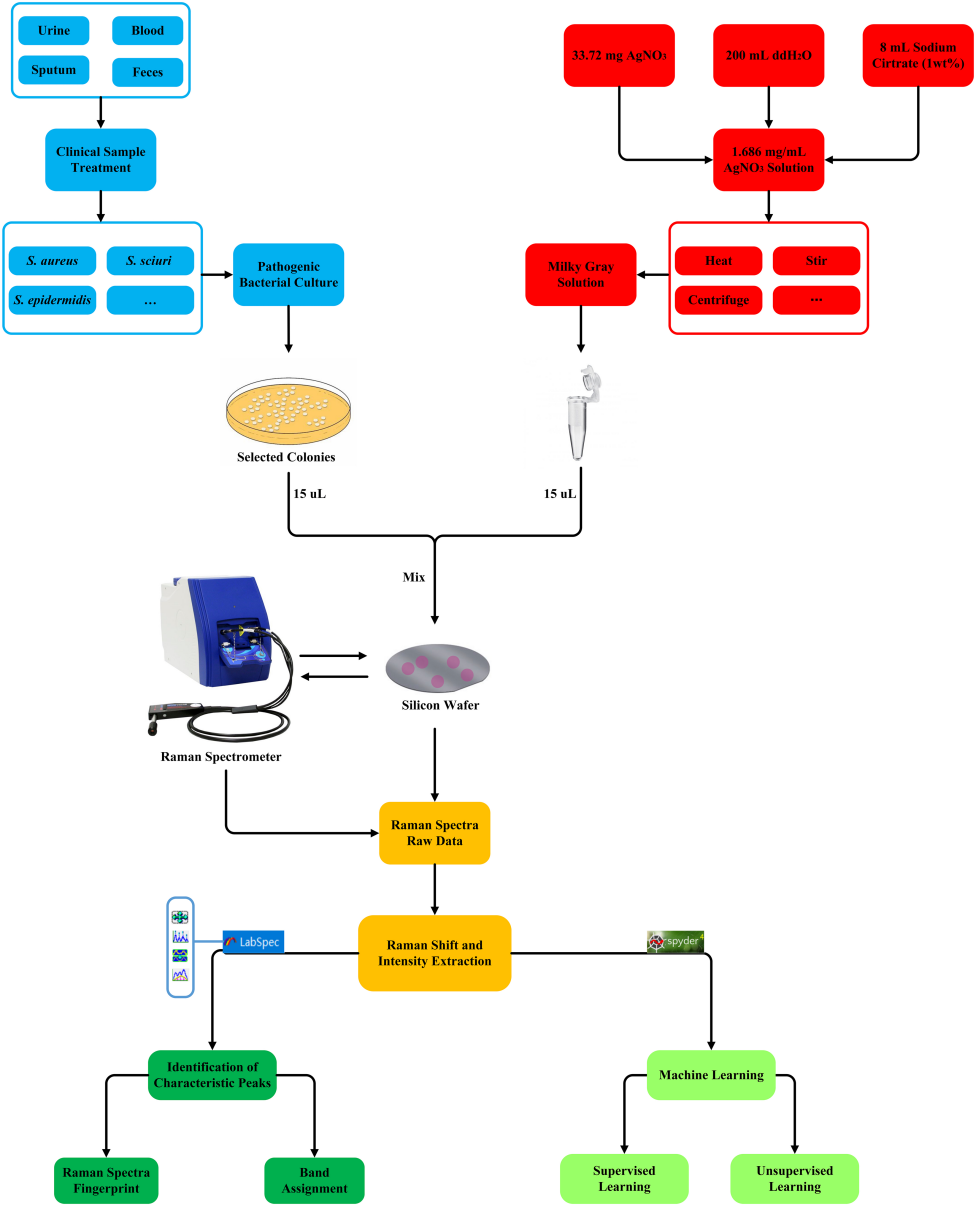
Schematic illustration of the generation and analysis of SERS spectra for the nine *Staphylococcus* species, which involve clinical sample collection and bacterial culture (blue square), AgNO_3_ silver nanoparticle solution preparation (red square), bacteria and AgNO_3_ solution mixture, Raman spectroscopy (yellow square), Raman spectra raw data pre-treatment (dark green square), and unsupervised and supervised machine learning algorithms (light green square).

Whether using supervised machine learning algorithms for regression, classification, or clustering, quantitative indicators for testing the effects of supervised machine learning models are inevitable and important. Therefore, all these algorithms were scored by the accuracy rate (ACC), recall rate (Recall), F1 score (F1-score), Matthews correlation coefficient (MCC), precision (Pre), and KAPPA coefficient (kappa). In order to compare the stability of the selected models, this study also used 5-fold cross-validation to evaluate the constructed models. Briefly, in each independent test, the whole data set was randomly divided into five subsets, four of which were used as training data sets while the remaining one was used as a verification dataset. The verification process could be repeated for different times to evaluate the stability of the model. The average accuracy of all verification segmentations was taken as the overall accuracy. In addition, in order to estimate a classifier’s ability to predict a certain sample at a specific threshold, receiver operating characteristic (ROC) curves were drawn for all the machine learning methods. The closer the ROC curve was to the upper left corner, the higher the true correct rate (TPR) of the test, and the lower the false correct rate (FPR). Therefore, the point on the ROC curve closest to the upper left corner had the largest sum of sensitivity and specificity. Finally, the classification and prediction results were visualized using a confusion matrix for CNN model. Rows corresponded to bacterial species identified by standard biochemical tests and MALDI-TOF MS (true class) while columns corresponded to bacterial identification predicted by the CNN algorithm. For a detailed procedure of machine learning analysis of *Staphylococcus* Raman spectra in this study, please refer to Figure 3.

## Results

### Raman Spectra for *Staphylococcus* Species

#### Average Raman Spectra

**Figure 4:**
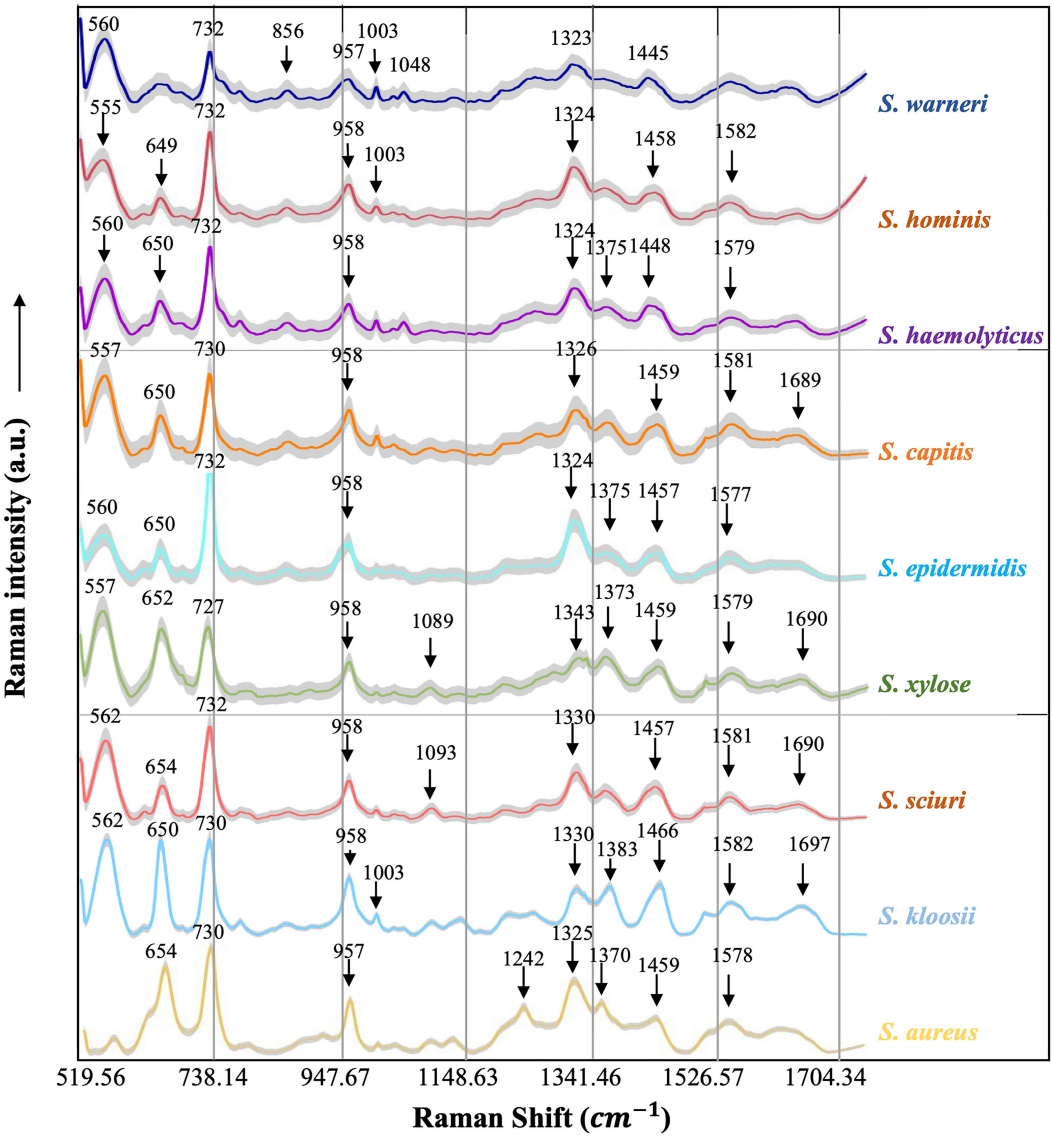
Averaged surface enhanced Raman spectra of nine clinical *Staphylococcus* species. For each Raman spectrum, multiple spectra were used, that is, *Staphylococcus aureus* (*n*=531), *Staphylococcus capitis* (*n*=282), *Staphylococcus epidermidis* (*n*=649), *Staphylococcus haemolyticus* (*n*=360), *Staphylococcus hominis* (*n*=550), *Staphylococcus kloosii* (*n*=80), *Staphylococcus sciuri* (*n*=70), *Staphylococcus warneri* (*n*=140), and *Staphylococcus xylose* (*n*=90). The *X*-axis represented Raman shift that ranged from 519.56 to 1,800.81cm^−1^ while the *Y*-axis represented Raman intensity in arbitrary unit (a.u.). For each spectrum, characteristic peaks were marked with black arrows and numbered with the corresponding Raman shift. Shadow region for each spectrum represented 20% of Raman shift SD.

Average Raman spectra with SE (shaded error bands) could clearly and quantitatively display the general trend and also reflect the data variance in the Raman spectra, which were present in Figure 4 for all the nine *Staphylococcus* species explored in this study. It was noteworthy that we used 20% of the SE at a given Raman shift for the visualization of the error bands. Since band shape was important for identifying characteristic peaks, we also used Savitzgy-Golay smoothing algorithm (also known as moving polynomial method) to preserve the band shape, which worked better than other methods such as moving average algorithm and Fourier filter that might lead to the loss of spectral information (Radzol et al., 2014).

#### Characteristic Peaks of Raman Spectra

Since each Raman spectrum contained multiple peaks, it was rather difficult to recognize the individual contributions of the numerous peaks. Thus, the average Raman spectra of the nine *Staphylococcus* species were analyzed by Gauss-Loren function *via* LabSpec software (Tagliaferro et al., 2020), through which characteristic peaks for each average Raman spectrum were identified and were marked with black arrows in Figure 4. According to previous studies, spectral peaks could be assigned to known metabolites; however, due to the complexity of the Raman spectra, identities of the metabolites could only be speculated (Nguyen et al., 2013). As for the Raman spectral results in this study, different peak combinations were observed for each *Staphylococcus* species (Table 1).

In specificity, all the species had prominent peaks at 555–562cm^−1^ (Guanine/Thymine/Uridine) except for *S. aureus* (Mert et al., 2015). As for Raman shift from 649 to 654 cm^−1^ (Guanine), all the species had characteristic peaks within this region except for *S. warneri* (Ahmed et al., 2013) while *S. warneri* had unique peak at 856cm^−1^ (Tyrosine; Chaturvedi et al., 2016). In addition, characteristic peaks at 727/730/732cm^−1^ (nucleic acids) were also present in all *Staphylococcus* species (Chao and Zhang, 2012). As for the C=C double bond (957 and 958cm^−1^), this characteristic peak was also identified in the Raman spectra of all *Staphylococcus* species (Ahmed et al., 2013). In the strains *S. warneri*, *S. hominis*, and *S. kloosii*, a unique characteristic peak 1,003cm^−1^ (C-H) was identified in all of them while other strains did not have this peak (Chaturvedi et al., 2016). It was also observed that P-O (1,048cm^−1^) was only present in *S. warneri* (Chen et al., 2015). In terms of phenylalanine (1,089, 1,093cm^−1^), only *S. xylose* and *S. sciuri* showed the reported peaks (Ahmed et al., 2013). In addition, all strains had the peak of adenine ring (1,323–1,330cm^−1^; Chisanga et al., 2018) while *S. haemolyticus*, *S. epidermidis*, *S. xylose*, *S. kloosii*, and *S. aureus* had a unique peak at 1,370–1,383cm^−1^ for the amide III (Perez-Guaita et al., 2016). It was noteworthy that *S. aureus* also had amide III at the characteristic peak 1,242cm^−1^ (Chisanga et al., 2018). The identification of peaks in the range of 1,445–1,466cm^−1^ in all the *Staphylococcus* species was indicative of N=N aromatic and aliphatic substance (Nguyen et al., 2013). In addition, guanine and adenine peaks (1,577–1,582cm^−1^) were observed in all the species except for *S. warneri* strains (Chisanga et al., 2018). Finally, the characteristic peak for the combination of C=O and C=C bonds in the range of 1,689–1,697cm^−1^ was seen in *S. capitis*, *S. xylose*, *S. sciuri*, and *S. kloosii* (Perez-Guaita et al., 2016).

### Unsupervised Machine Learning Methods

**Figure 5:**
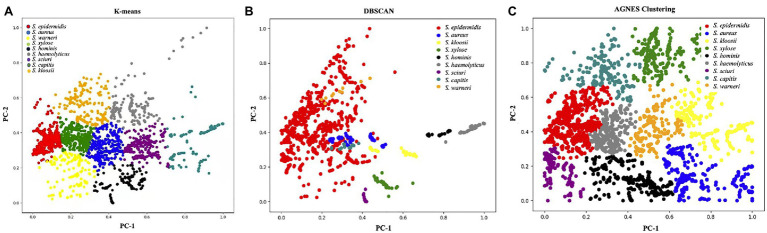
Clustering results of nine *Staphylococcus* species *via* **(A)** K-means, **(B)** density-based spatial clustering of applications with noise (DBSCAN), and **(C)** agglomerative nesting (AGNES). The PCA score plot showed the two directions of largest variance in the data and provided valuable insights into the nature of the surface enhanced Raman spectra of *Staphylococcus* species. Each color corresponded to one group of *Staphylococcus* species as denoted in the figure legend.

Unsupervised learning algorithms aim to seek the representations of a mixed dataset by splitting the data into well-separated groups called clusters. Thus, unsupervised methods are mainly used for clustering data without *a priori* knowledge (Sikirzhytski et al., 2010). In this study, we employed three commonly used unsupervised machine learning algorithms for clustering surface enhanced Raman spectra of *Staphylococcus* species, which included K-means, DBSCAN, and AGNES. K-means partitions data into *k* distinct clusters based on distance to the centroid of a cluster, which have been successfully applied to the analysis of Raman spectra from biological samples such as breast cancer (Kothari et al., 2021), colonic cancer (Beljebbar et al., 2009), and macromolecules (Pahlow et al., 2018). As for the DBSCAN algorithm, it is a density-based clustering that looks for high-density areas and extends clusters from them (Guyeux et al., 2019). Thus, the pre-set number of clusters is not required. In terms of AGNES, it uses hierarchical agglomerative approach to divide a dataset into clusters *via* successive fusions of the individual objects (Oyelade et al., 2016). However, the three methods are rarely used for Raman spectral analyses. In this study, all the clustering results were visualized in Figure 5 *via* Python scikit-learn library, from which a clear picture of clustering effects could be observed. In order to obtain a qualitative comparison of the performance of the three methods, Rand Index, a metric for the assessment of cluster algorithm performance, was calculated (Rand, 1971). According to the result, DBSCAN had the highest score of 0.9733 while Rand indices for K-means and AGNES are 0.933 and 0.9291, respectively. In sum, the result suggested that the discrimination of *Staphylococcus* species *via* unsupervised machine learning analysis of surface enhanced Raman spectra was plausible, which had the potential to be applied in clinical settings.

### Supervised Machine Learning Methods

#### Comparison of 10 Supervised Machine Learning Algorithms

Supervised learning is to use an algorithm to learn the mapping function *Y*=*f*(*X*), where *X* is an input variable while *Y* is an output variable. The purpose of the learning process is to use unknown input data *X* to accurately predict its output *Y*. Briefly, supervised learning algorithms aim to establish a correlation between known input variables and dependent variables (labels) *via* data training in order to predict the outcomes of new input variables (Xu and Jackson, 2019).

In this study, we compared 10 commonly encountered methods, which were KNN, DT, RF, GB, SVM, AdaBoost, GNB, QDA, CNN, and LSTM, in terms of their capacities in the analysis of *Staphylococcus* Raman spectral data. Through calculating and comparing the machine learning scores, that is, ACC, Recall, F1-score, MCC, Pre, and KAPPA, we revealed that the deep learning algorithm CNN had the best prediction accuracy (98.21%), together with the largest AUCs (99.93%). Recently, Ho et al. (2019) also compared the performance of classic machine learning methods, that is, logistic regression (LR) and SVM, with the deep learning algorithm CNN, which showed a similar result. In particular, LR and SVM achieved accuracies of 75.7 and 74.9%, respectively, while CNN had an average isolate-level accuracy of 82.2±0.3% (Ho et al., 2019). Except for CNN, another deep learning algorithm LSTM that was rarely used for Raman spectral analysis also performed well with high ACC (94.33%) and AUC (99.83%) values. In addition, classic methods, such as KNN, RF, DT, and GB also achieved good prediction accuracies while SVM, AdaBoost, QDA, and GNB were not recommended for the analysis of Raman spectra of *Staphylococcus* species. Predication performance of the 10 supervised machine learning methods is present in Table 2.

**Table 2:** Comparison of 10 machine learning algorithms in terms of their capacities in the analysis of *Staphylococcus* Raman spectral data.

Classifier	ACC	Pre	Recall	F1	KAPPA	MCC	5-fold CV	AUC
CNN	98.21	98.61	95.83	98.62	N/A	95.32	97.44	99.93
LSTM	94.33	91.61	90.03	91.67	89.47	89.85	92.5	99.83
KNN	96.22	96.2	94.05	96.19	95.25	95.26	93.9	98.03
RF	94.55	94.53	90.32	94.45	93.14	93.16	91.89	97.01
DT	90.32	90.3	88.13	90.32	87.87	87.89	88.7	94.59
GB	94.55	94.55	90.66	94.41	93.13	93.16	92.47	89.05
SVM	34.95	94.93	15.81	24.28	9.97	14.26	34.02	89.05
AdaBoost	27.24	27.3	20.29	16.69	8.04	10.32	31.51	73.81
QDA	31.77	31.6	28.92	25.83	13.68	15.21	37.35	61.01
GNB	13.46	13.43	32.9	9.35	6.85	8.7	14	56.22

Since CNN achieved the highest prediction accuracy and largest AUC value, we investigated into data analysis procedure in order to explain how the algorithm was refined in this study. In specificity, For the CNN model, it can automatically extract features from things without artificial intervention, avoiding complex data preprocessing procedures. In addition, the convolutional layer and the pooling layer in the CNN algorithm are alternately applied, using different convolution kernels and the entire range of data for convolution. Thus, the algorithm greatly simplified the amount of data, improved computing efficiency and robustness, and completed nonlinear multi-classification through the fully connected layer task. In this study, we used the classic deep learning model LeNet-5, with six convolutional layers, three pooling layers, and two fully connected layers, while the size of the convolution kernel is set to 3*1. Then, each Raman spectrum was input into the CNN in the form of one-dimensional data. The Raman shift ranged from 519.56 to 1,800.81cm^−1^, leading to the generation of a total of 667 Raman shifts. ReLU activation function was used to avoid the problems of gradient explosion and gradient disappearance, which speeded up the model convergence. The Adam loss function was used to avoid the model from falling into a local minimum. Since the recognition target format is in One-Hot Encoding form, categorical_crossentropy was used as the loss. In order to facilitate the orderly linking of neurons in the network, Flatten Layer was used to stretch the data into one column, followed by the Softmax output layer in order to realize multi-class identification of pathogenic bacteria samples.

**Figure 6:**
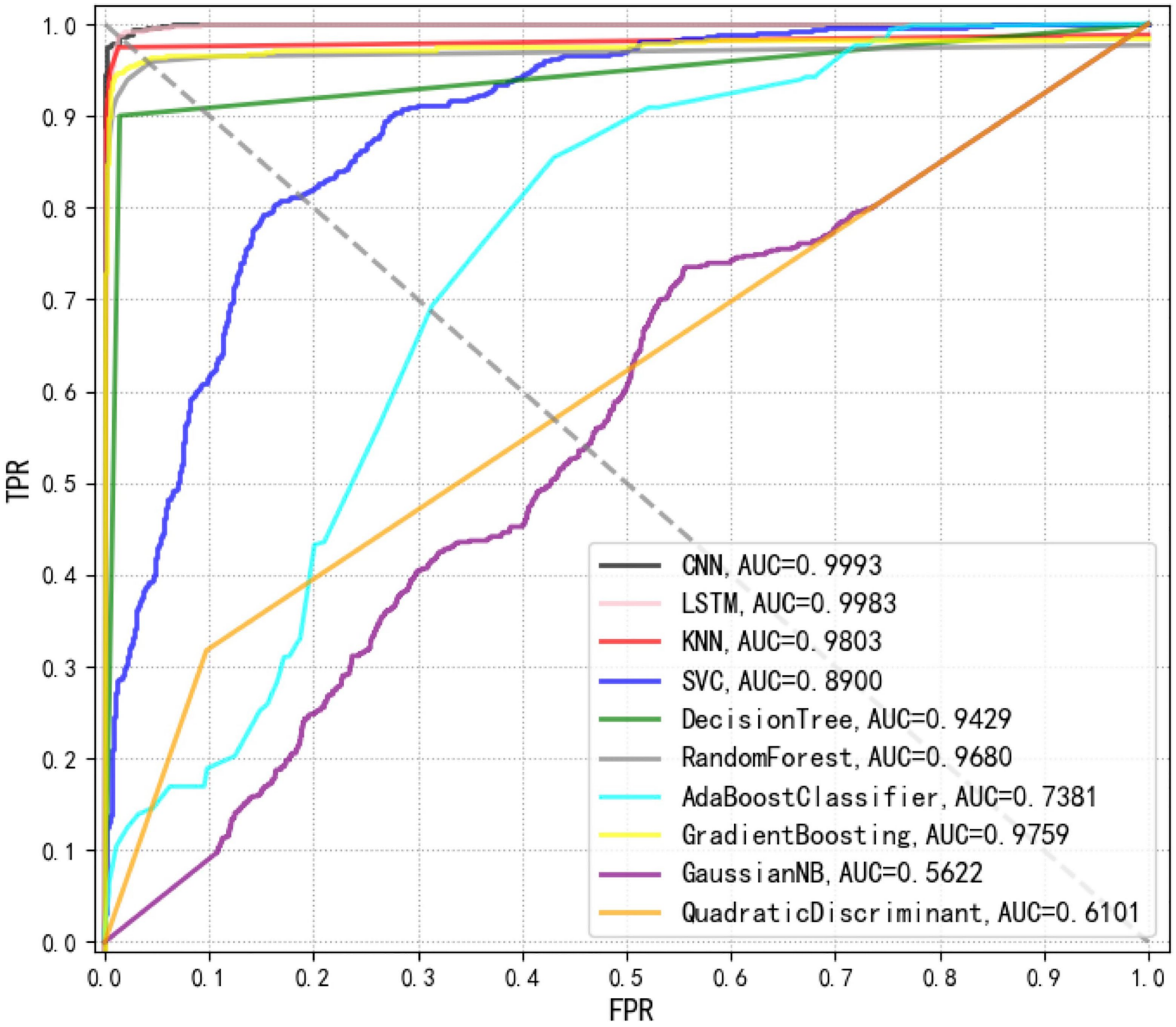
Comparison of receiver operating characteristic (ROC) curves used for the evaluation of the performance of 10 supervised machine learning algorithms. The closer the ROC curve is to the upper left corner, the higher the true correct rate (TPR) and the lower the false correct rate (FPR) of the test. According to the comparison, CNN achieved the best performance [area under curve (AUC)=0.9993] than all other algorithms.

Receiver operating characteristic curves compare sensitivity and specificity across a range of values for the ability of supervised machine learning methods to predict a dichotomous outcome while the area under the ROC curves (AUCs) mean overall accuracies in distinguishing *Staphylococcus* species among each other (Florkowski, 2008). Thus, ROC curves are a graphical demonstration of true positives and false-positives across a range of cut-offs. In this study, we compared the ROC curves of 10 supervised machine learning methods, together with the corresponding AUCs, which clearly showed that the top three methods with the best performances were CNN, LSTM, and KNN (Figure 6).

#### Confusion Matrix for CNN Algorithm

**Figure 7:**
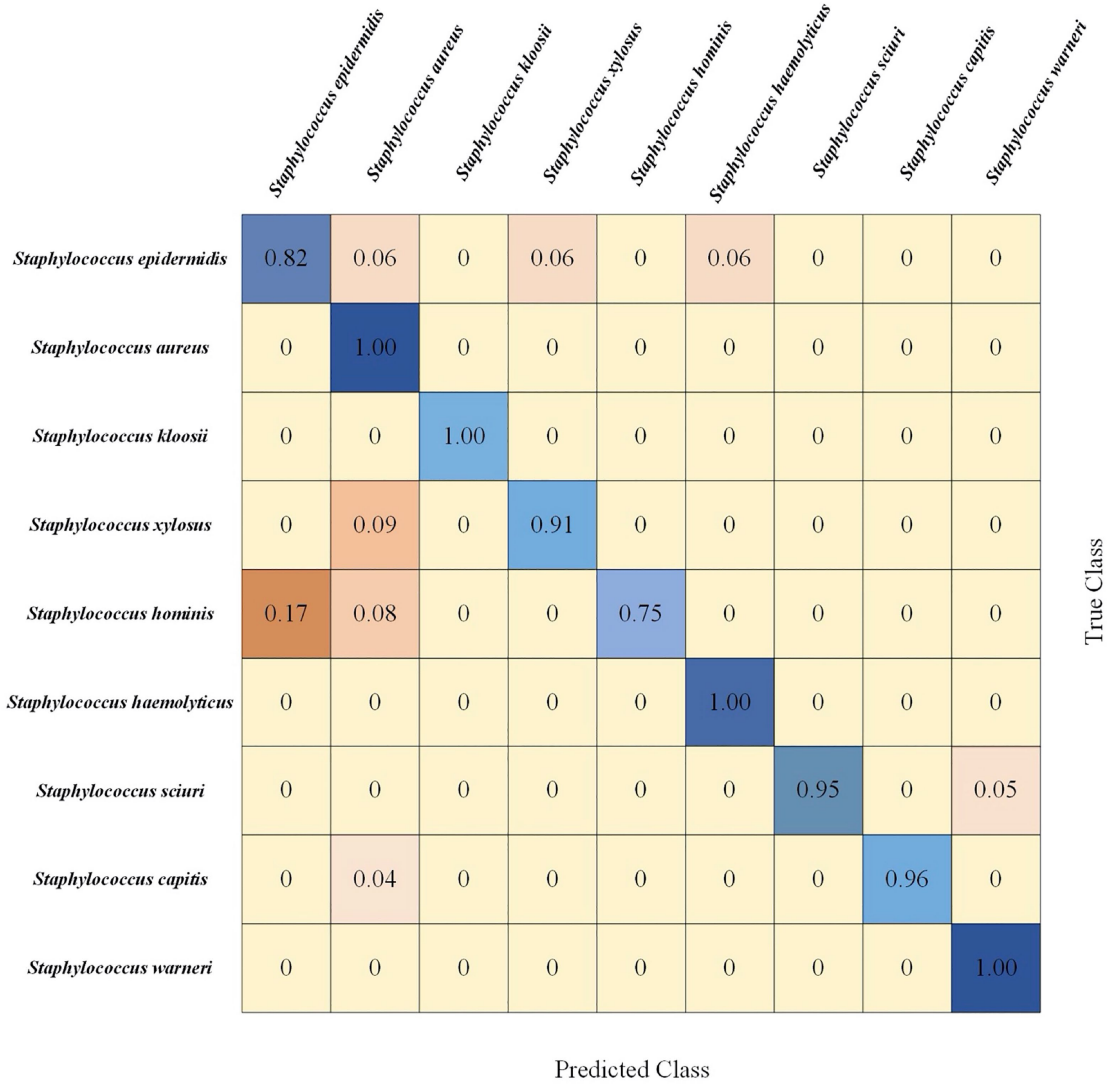
Confusion matrix of 5-fold cross-validated *Staphylococcus* species identification *via* CNN model. Rows corresponded to bacterial species identified by standard biochemical tests and matrix-assisted laser desorption/ionization-time of flight (MALDI-TOF) mass spectrometry (MS; true class) while columns corresponded to bacterial identification predicted by the CNN algorithm (predicted class). Numbers in the confusion matrix stood for the percentage of correctly classified (diagonal) or mis-classified (off-diagonal) spectra, respectively.

Confusion matrix is a table that describes the classification results in detail, including true class and predicted class. Since CNN performed best in terms of *Staphylococcus* species prediction in this study, we calculated the corresponding confusion matrix of binary classification, which provided further classification details (Figure 7). In this matrix, the vertical axis denoted the true classes (actual classes of samples *via* standard biochemical tests and MALDI-TOF MS) while the horizontal axis represented the predicted classes. In addition, we classified the samples into nine categories, that is, nine *Staphylococcus* species. Using the confusion matrix, we evaluated the performance of the CNN model on each species. According to the matrix, the CNN model had the lowest accuracy for predicting *S. hominis* (75%) that was misclassified as *S. epidermidis* in 17% and as *S. aureus* in 8% of all the cases. On the other hand, the model had 100% accuracy in predicting the species *S. aureus*, *S. kloosii*, *S. haemolyticus*, and *S. warneri*.

## Discussion

Conventional methods such as medium culture and biochemical reactions for bacterial species differentiations and phenotype profilings are sometimes laborious and time-consuming (Palomino, 2005) while commonly used molecular methods like PCR and enzyme-linked immunosorbent assay (ELISA) either require specially designed primers or have comparatively high false positive rates due to the instability of some antibodies (Sakamoto et al., 2017). As for the newly developed high-throughput sequencing technology, although the sequencing costs have dropped significantly, the complex data analysis pipeline and traditional clinical laboratory procedures somewhat restrict its wide application in clinical diagnosis (Bertelli and Greub, 2013). Compared to the above-mentioned methods, Raman spectroscopy is a fast, sensitive, low-cost, label-free, and non-destructive microbial detection and identification technique (Maruthamuthu et al., 2020), which has great potential in facilitating the improvement of the clinical diagnosis (Wang et al., 2021). In fact, a variety of studies has already used Raman spectroscopy for the identification of *Staphylococcu*s species. For example, Samek et al. (2008) analyzed the Raman spectra of *S. epidermidis* related to medical device-associated infections, based on which different *S. epidermidis* clones were discriminated *via* combinational analyses of characteristic peaks. Later, Rebrošová et al. (2017) used three supervised learning methods, LDA, 1NN, and SVM, to analyze 16 *Staphylococcus* strains in order to differentiate between *S. aureus* and *S. epidermidis* strains.

Due to the low signal-to-noise ratio of Raman spectroscopy from bacterial species and phenotypes, it is difficult to quickly and accurately characterize these biological samples (Zhu et al., 2019). Thus, SERS was developed to enhance Raman scattering effects. For example, Chen et al. (2019) performed the SERS through positively charged silver nanoparticles and successfully identified MRSA *S. aureus* with almost 100% accuracy. However, it is noteworthy that silver nanoparticles are toxic to bacterial organisms, which would affect the experimental results and cause the variation of Raman spectra (Cui et al., 2015). In this study, we used the same amount of AgNPs (15μl) to mix with the 15μl solution of selected colonies of *Staphylococcus* species, which will generate similar impacts on bacterial metabolism and physiology. In addition, for the same species, we have multiple strains for Raman spectral analyses, which would be considered as biological replicates and reduce the intra-group variations. During the computational analysis of Raman spectra, the whole spectra rather than specific peaks were analyzed, which would also reduce the influences of small variations in the fingerprinting spectra caused by the toxicity of silver nanoparticles. Characteristic peaks could not only reflect the unique patterns of Raman spectra but also correspond to specific compositions of bacterial species (de Siqueira E Oliveira et al., 2020). Thus, it was biologically meaningful to analyze the characteristic peaks of Raman spectrum for each *Staphylococcus* species. Currently, a variety of computational tools like LabSpec 6 (HORIBA Scientific, Japan), and algorithms like occlusion-based Raman spectra feature extraction (ORSFE) have been developed for identifying characteristic peaks in Raman spectra (Lu et al., 2020), all of which could perform well on Raman spectra for feature extraction.

In addition, Raman spectra are rather complex and classical linear methods are no longer sufficient for data processing (Lussier et al., 2020). Thus, advanced computational methods are essential in processing these sophisticated data. In this study, we compared the capacities of machine learning methods in discriminating and predicting bacterial species through the analyses of the SERS spectra of nine *Staphylococcus* species. For unsupervised machine learning analysis of Raman spectra, previous studies have successfully applied PCA and hierarchical cluster analysis (HCA) on bacterial pathogens such as meningococcus and mycobacteria (Harz et al., 2009; Stöckel et al., 2015). However, clustering algorithms, such as K-means, DBSCAN, and AGNES are rarely used. Our quantitative comparison shows that DBSCAN has the best clustering performance for the SERS spectra of nine *Staphylococcus* species. In particular, DBSCAN is a very typical density clustering algorithm. Compared with K-means and AGNES that are generally suitable for convex sample sets, DBSCAN can be applied to both convex and non-convex sample sets. The significant advantage of the DBSCAN algorithm is that the clustering speed is fast and it can effectively deal with noisy points and find spatial clusters of arbitrary shapes. Raman spectroscopy data have different signal intensities at different Raman shifts. In this study, Raman spectra were first passed through data preprocessing procedures, including curve smoothing, baseline correction, polynomial fitting, and intensity normalization. The DBSCAN algorithm then calculates the average Euclidean distance between the Raman shift and the signal intensity of each sample and each cluster, and selects the smallest distance to divide the clusters. Parameters were manually adjusted and the key parameters used in the DBSCAN algorithm were set to min_sample=9 and eps=0.7.

Besides discriminating bacterial species into different groups, we also compared supervised learning algorithms for the predictions of bacterial species. A variety of supervised machine learning methods have been used for Raman spectral analyses, such as SVM (Moawad et al., 2019), RF (Ren et al., 2017), CNN (Wang et al., 2020), KNN (Uysal Ciloglu et al., 2020), and DT (Uysal Ciloglu et al., 2020), etc. Although many supervised learning methods have been used for various bacterial species, which leads to comparatively high prediction accuracies, there are rarely comparative studies of the performance of supervised machine learning methods in surface enhanced Raman spectral analysis. In this study, we compared 10 commonly used supervised learning algorithms for their capacities in Raman spectral analysis, among which CNN and LSTM topped other algorithms and performed the best. Other methods, such as KNN, RF, DT, and GB also achieved high level of prediction accuracies but did not surpass CNN and LSTM. In particular, Raman spectroscopy generates fingerprinting spectra that are difficult to avoid the influence of various objective factors during the acquisition process. Thus, it is necessary to clean and preprocess the spectral data. After that, we compared eight traditional supervised learning algorithms and two deep learning algorithms. According to the results, KNN has the highest accuracy among traditional machine learning algorithms, with an accuracy of 96.22%, which can effectively distinguish nine different *Staphylococcus* species. The accuracy of RF and DT is slightly lower than that of KNN, and the accuracy is up to 90%.

For those who are not familiar with the preprocessing process of Raman spectroscopy of pathogenic bacteria, the process of spectral preprocessing is a complicated process. Thus, in this study, we applied two deep learning methods, CNN and LSTM, to remove complex pre-processing procedures through automatically extracting spectral features based on the construction of convolutional layers, pooling layers, fully connected layers, and activation functions. The accuracy rates of CNN and LSTM reached 98.21 and 94.33% while the AUC values reached 99.93 and 99.83%, respectively. In order to reflect the generalization ability of the deep learning algorithm, this study also used a 5-fold cross-validation method to objectively evaluate the robustness of the model. The cross-validation results for CNN and LSTM reached 97.44 and 92.5%, respectively, which showed that deep learning algorithms had strong classification and prediction ability in the identification of bacterial pathogen identification through Raman spectra. However, these results were only based on laboratory cultures of *Staphylococcus* species. Further studies will be focusing on direct discrimination and prediction of bacterial pathogens from clinical samples, such as sputum, urine, and blood, etc., which, despite a very challenging question, will greatly facilitate the real world applications of Raman spectroscopy in clinical settings.

## Conclusion

Raman spectroscopy has been widely used in the diagnosis of bacterial pathogens in terms of species differentiation, antibiotic resistance detection, and virulence factor identification (Rebrošová et al., 2017). In this study, we explored both unsupervised and supervised machine learning algorithms in terms of their capacities to discriminate and predict pathogenic *Staphylococcus* species *via* SERS spectra. According to the results, DBSCAN showed the best clustering effect while CNN was the best prediction model for the SERS spectra of nine *Staphylococcus* species. However, there are many machine learning algorithms that have not been explored, which may be appropriate for the analysis of Raman spectra and worthy of further investigation. Moreover, machine learning algorithms should also be applied for more sophisticated situations, such as identifying bacterial species directly from clinical samples, rather than relying on isolated and cultured bacterial colonies. Specialized Raman spectral database for clinically important bacterial pathogens should also be constructed, which could greatly improve the implementation of Raman spectroscopy in clinical settings. Taken together, this study showed the great potential of Raman spectroscopy in culture-free pathogen identification that could facilitate the fast and accurate clinical diagnosis and swift control of infectious diseases.

## Data Availability Statement

The original contributions presented in the study are included in the article/Supplementary Material, further inquiries can be directed to the corresponding authors.

## Author Contributions

LW conceived and designed the experiments. LW, Z-BZ, and BG contributed to project administration. J-WT, Q-HL, X-CY, P-BW, XL, and X-XK carried out the computational and experimental work. LW, J-WT, Q-HL, Y-CP, and XL wrote and revised the manuscript. LW and BG provided platform, resources, and student supervision. All authors contributed to the article and approved the submitted version.

## Conflict of Interest

The authors declare that the research was conducted in the absence of any commercial or financial relationships that could be construed as a potential conflict of interest.

## Publisher’s Note

All claims expressed in this article are solely those of the authors and do not necessarily represent those of their affiliated organizations, or those of the publisher, the editors and the reviewers. Any product that may be evaluated in this article, or claim that may be made by its manufacturer, is not guaranteed or endorsed by the publisher.

## Supplementary Material

Supplementary Figure 1 Dot matrix plot of the characteristic peaks in the nine *Staphylococcus* species that was identified *via* LabSpec 6.
