# Supplementary figures and images for: Comparative Analysis of Machine Learning Algorithms on Surface Enhanced Raman Spectra of Clinical Staphylococcus Species
Source: Front Microbiol. 2021 Aug 31;12:696921. doi: 10.3389/fmicb.2021.696921 (PMC8439569; doi:10.3389/fmicb.2021.696921)

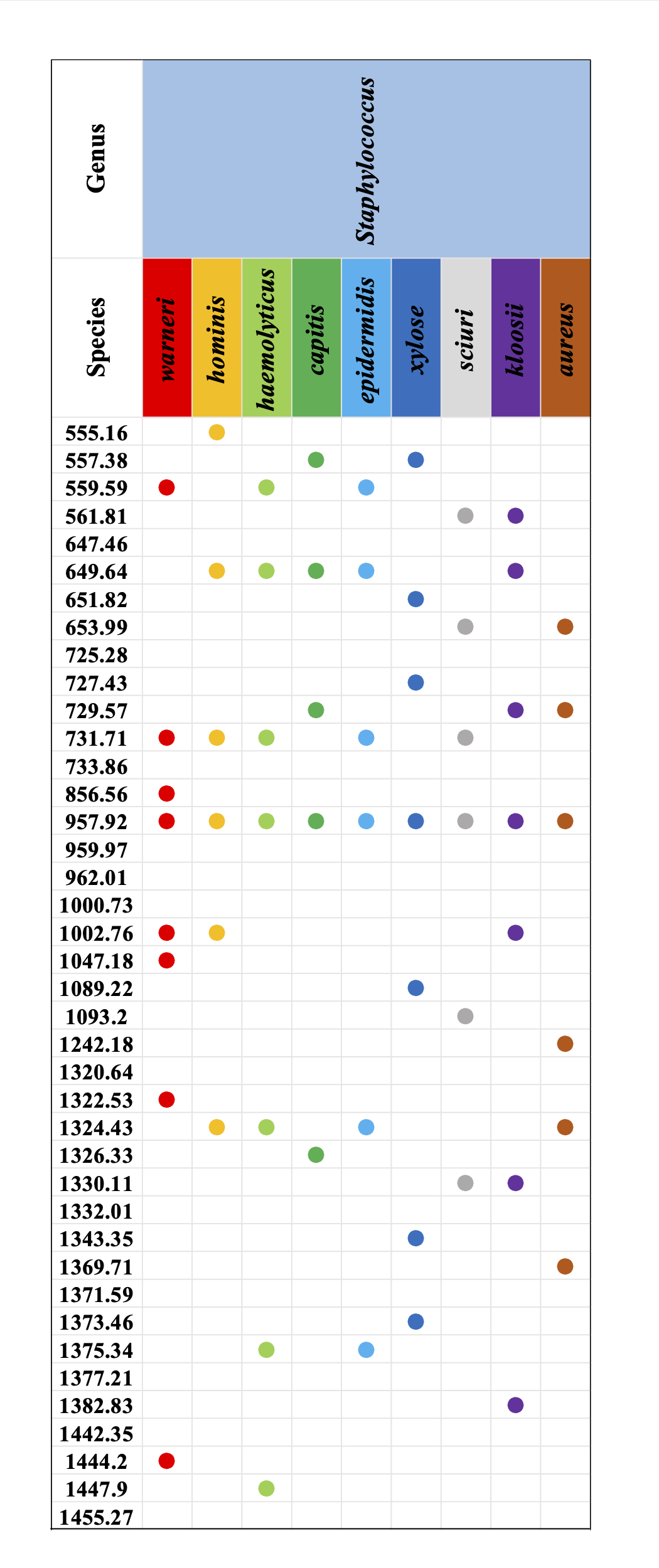

Supplement: Supplementary Figure 1 — Dot matrix plot of the characteristic peaks in the nine Staphylococcus species that was identified via LabSpec 6. [file Image_1.JPEG]
